# Increasing Hybrid Rice Yield, Water Productivity, and Nitrogen Use Efficiency: Optimization Strategies for Irrigation and Fertilizer Management

**DOI:** 10.3390/plants13121717

**Published:** 2024-06-20

**Authors:** Haijun Zhu, Xiaoe He, Xuehua Wang, Pan Long

**Affiliations:** Key Laboratory of Ministry of Education for Crop Physiology and Molecular Biology, Hunan Agricultural University, Changsha 410128, China; 15211104718@163.com (H.Z.); hexiaoe2022@163.com (X.H.)

**Keywords:** irrigation strategies, fertilizer management, rice yield, water productivity, nutrient utilization

## Abstract

Water and fertilizer are crucial in rice growth, with irrigation and fertilizer management exhibiting synergies. In a two-year field study conducted in Yiyang City, Hunan Province, we examined the impact of three irrigation strategies—wet-shallow irrigation (W1), flooding irrigation (W2), and the “thin, shallow, wet, dry irrigation” method (W3)—in combination with distinct fertilizer treatments (labeled F1, F2, F3, and F4, with nitrogen application rates of 0, 180, 225, and 270 kg ha^−1^, respectively) on rice yield generation and water–fertilizer utilization patterns. The study employed Hybrid Rice Xin Xiang Liang you 1751 (XXLY1751) and Yue Liang you Mei Xiang Xin Zhan (YLYMXXZ) as representative rice cultivars. Key findings from the research include water, fertilizer, variety, and year treatments, which all significantly influenced the yield components of rice. Compared to W2, W1 in 2022 reduced the amount of irrigation water by 35.2%, resulting in a 42.0~42.8% increase in irrigation water productivity and a 25.7~25.9% increase in total water productivity. In 2023, similar improvements were seen. Specifically, compared with other treatments, the W1F3 treatment increased nitrogen uptake and harvest index by 1.4–7.7% and 5.9–7.7%, respectively. Phosphorus and potassium uptake also improved. The W1 treatment enhanced the uptake, accumulation, and translocation of nitrogen, phosphorus, and potassium nutrients throughout the rice growth cycle, increasing nutrient levels in the grains. When paired with the F3 fertilization approach, W1 treatment boosted yields and improved nutrient use efficiency. Consequently, combining W1 and F3 treatment emerged as this study’s optimal water–fertilizer management approach. By harnessing the combined effects of water and fertilizer management, we can ensure efficient resource utilization and maximize the productive potential of rice.

## 1. Introduction

With the growth of the population and the decrease in arable land, food security has emerged as a global concern. As the world’s largest producer and consumer of rice, China undertakes a crucial responsibility in rice production [1]. Hybrid rice, a significant achievement of Chinese agricultural technology, boasts a yield far higher than traditional rice varieties, which is pivotal in ensuring China’s food security [2,3]. However, the high yield of hybrid rice also raises a series of issues, such as high water consumption and low fertilizer utilization efficiency [4]. Therefore, improving water and fertilizer utilization efficiency while maintaining the high yield of hybrid rice and reducing the environmental burden has become an important topic in current agricultural scientific research [5].

Water resources are an indispensable condition for agricultural production [6]. In 2021, Chinese agricultural water consumption amounted to 364.43 billion cubic meters, accounting for 61.5% of the country’s total water consumption [7]. Rice, in particular, is the most water-consuming crop in agricultural production, utilizing 70% of the agricultural water consumption [8]. Over 95% of rice irrigation in China relies primarily on traditional flooded irrigation methods. However, this irrigation method consumes significant water, with approximately half lost through various means [9]. According to research, the current irrigation water consumption for paddy fields in southern China exceeds 9000 m^3^ per hectare. In contrast, due to low rainfall in some northern regions, the irrigation water amount surpasses 15,000 m^3^ per hectare [10]. This is far higher than the irrigation water required for local rice production, indicating a severe waste of water resources. Due to the lack of effective water-saving irrigation techniques and management, a significant portion of irrigation water is lost during field production [11]. Therefore, developing water-saving agriculture and researching high-yield and efficient water-saving irrigation techniques for rice is a significant strategic need in China. Approximately 95% of China’s rice paddies are suitable for water-saving irrigation [12]. By fully adopting water-saving irrigation methods, China’s rice production could increase by 5.4% to 6.9% while saving 22.1% to 26.4% of irrigation water [13]. Researching water-saving irrigation technologies to grow rice with less water and improve water productivity, thereby achieving sustainable irrigation, is significant for stabilizing China’s rice production safety and optimizing water resource utilization.

Notably, fertilizer is also an essential nutrient for the growth of hybrid rice [14]. China occupies only 9% of the world’s cultivated land area. Yet, its fertilizer application accounts for one-third of the global total [15]. The fertilizer application per hectare of crops in China reaches 506.11 kg, which is 2.05 times that of the UK, 3.69 times that of the US, and 9.45 times that of Australia, significantly higher than the levels seen in developed countries worldwide [16]. This high usage is primarily attributed to the pressure to increase rice production, low soil fertility, high utilization intensity of cultivated land, large-scale agricultural production, and outdated fertilization techniques. Due to outdated fertilization techniques and a limited range of fertilizer types, the utilization rate of fertilizers remains low in practical agricultural production, resulting in significant fertilizer runoff into the environment. This leads to a waste of resources and causes environmental pollution [17]. In order to overcome these problems, several nitrogen-efficient management strategies, such as the balanced application of nitrogen fertilizer, optimized and reduced nitrogen management, organic fertilizers, and the adoption of controlled-release fertilizers, have been widely carried out [18,19].

Water and fertilizer are two fundamental factors for rice growth, which significantly impact crops and the environment and interact and restrict each other [20]. Studies have shown that the application effect of suitable water and fertilizer coupling technology is far greater than the single application effect of the two factors. Reasonable water and fertilizer regulation can promote the release of soil nutrients in paddy fields, enhance root activity, and enable more soil water and nutrients to enter the roots, which are then transported to the aboveground parts [21,22]. This helps to increase the transpiration rate, enhance net photosynthetic production, and facilitate the accumulation of rice dry matter, thereby increasing yield. Meanwhile, due to enhanced root activity, nutrients’ absorption and transformation capacity is relatively increased, improving the utilization rate of elements such as nitrogen, phosphorus, and potassium [23].

The middle and lower reaches of the Yangtze River are important rice cultivation areas, constituting the most intensively rice-farmed region in China and one of the most crucial rice production regions globally [9]. Upon investigation, it is discovered that rice growers in the middle and lower reaches of the Yangtze River have a tradition of cultivating rice under flood conditions and applying excessive fertilization [24,25]. This study aims to evaluate the interaction effects of three irrigation systems (wet-shallow irrigation, flooding irrigation, and “thin, shallow, wet, dry” irrigation) and four fertilizer management strategies (F1, F2, F3, and F4, corresponding to nitrogen application rates of 0, 180, 225, and 270 kg ha^−1^, respectively) on rice yield, fertilizer utilization efficiency, and WP, and to elucidate the possible reasons for any observed differences. This is crucial for rice production, water-conserving irrigation in the rice planting area in the middle and lower Yangtze River basin, and promoting the sustainable development of paddy fields in China.

## 2. Results

### 2.1. Grain Yield and Yield Components in Response to Irrigation Water and Fertilizer

Both water and fertilizer treatments significantly increased rice yield as primary effects (Table 1). We found a notable interaction between water and fertilizer. This interaction had a similar impact on the two rice varieties tested over two years (i.e., there were no significant interactions among water, fertilizer, and variety, neither were there significant interactions among water, fertilizer, and year). This suggests that when water and fertilizer treatments are combined, the increase in yield is even more substantial. Additionally, there were differences in rice yield between years, which can be attributed to significant interactions between variety and water treatment, as well as variety and fertilizer. Indeed, water, fertilizer, variety, and year treatments significantly influenced rice yield components (including panicles, spikelet per panicle, spikelet filling, and grain weight).

### 2.2. The Accumulation of N, P, and K

Irrigation methods and nitrogen fertilizer management have significant or highly significant effects on the accumulation of nitrogen, phosphorus, and potassium in vegetative organs, ear parts, and mature plants during the heading and maturity stages (Table 2, Table 3 and Table 4). Additionally, both factors show significant or highly significant interaction effects on the accumulation of nitrogen, phosphorus, and potassium in various organs during the heading and maturity stages. Focusing on nitrogen accumulation in plants as an illustrative example, since the accumulation of nitrogen, phosphorus, and potassium exhibits similarity across different treatments, Table 1 indicates notable differences in nitrogen accumulation among varieties and between years for straw, grain, and the total plant. Specifically, XXLY1751 shows significantly higher nitrogen accumulation compared to YLYMXXZ. Regarding yearly variation, 2023 demonstrates significantly higher nitrogen accumulation than 2022. Regarding the distribution of nitrogen among organs, straw accounts for an average of 57.86%, and grain accounts for 42.14%. Significant differences in nitrogen accumulation are observed among various water and fertilizer treatments.

### 2.3. N Harvest Index and Nitrogen Use Efficiency

Water and fertilizer treatments significantly affected the N harvest index (NHI) and N use efficiency (NAE), partial factor productivity of applied N (NPFP), and apparent recovery efficiency of N (NRE). At the same time, varietal differences and yearly variations also had a notable impact on these indicators, except NAE for varieties and NRE for yearly variations (Table 5). A significant interaction was observed between F×W for the NHI, but this interaction was not significant for NAE, NPFP, and NRE. Moreover, nitrogen application had a more pronounced effect on nitrogen use efficiency than irrigation methods. Compared to W2, W1 resulted in significant increases in NHI and NAE, NPFP, and NRE, with increases of 3.99%, 4.66%, 4.15%, and 3.03% in 2022, and 3.42%, 8.75%, 4.08%, and 3.58% in 2023, respectively. Among fertilizer treatments, F3 significantly increased NHI, NAE, NPFP, and NRE compared to F4, with notable increases of 3.96%, 71.14%, 30.86%, and 21.28% in 2022, and 3.86%, 59.90%, 29.84%, and 21.11% in 2023.

### 2.4. Water Productivity

**Figure 1:**
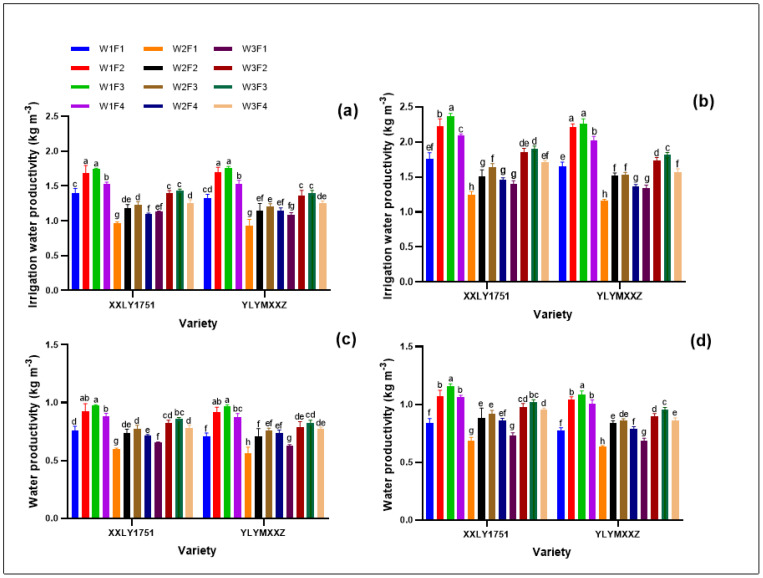
Irrigation water productivity in 2022 (**a**) and 2023 (**b**), and water productivity in 2022 (**c**) and 2023 (**d**). Different lowercase letters denote statistical differences between treatments of each variety according to the LSD test (0.05).

The water productivity (WP) of rice under different water and fertilizer management in 2022 and 2023 is illustrated in the accompanying figure (Figure 1). Compared to W2, W1 significantly enhanced the irrigation water productivity (IWP) and the overall WP. Specifically, in 2022, there was an increase of 24.04% to 40.99% for IWP and 15.61% to 4.58% for WP. Similarly, in 2023, the improvements ranged from 24.22% to 42.41% for IWP and 13.33% to 22.39% for WP. Under the same irrigation treatment, when compared to the N1 treatment, the IWP and WP were elevated in the N2, N3, and N4 treatments. Moreover, W1F2 and W1F3 treatments contributed to higher IWP and WP, with W1F3 being the most effective. In 2022, under W1F3, the IWP and WP were, respectively, higher than other treatments by 3.91% to 78.93% and 2.83% to 64.56%. Similarly, in 2023, the increases ranged from 4.47% to 7.11% for IWP and a remarkable 105.61% to 85.19% for WP.

### 2.5. Optimization of Water and Fertilizer Management Based on Yield

**Figure 2:**
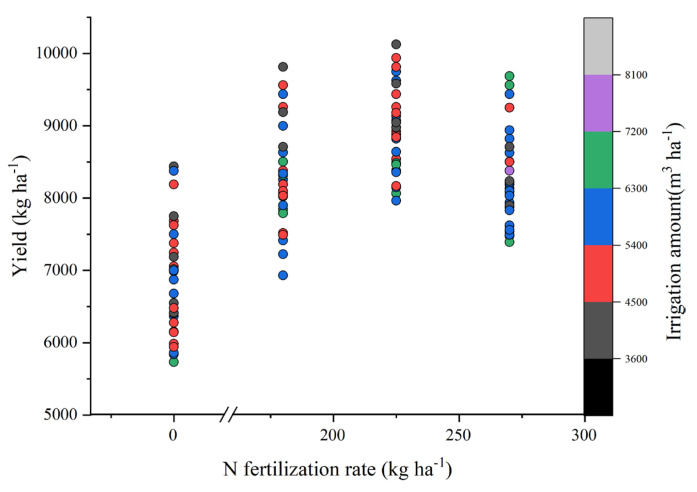
Relationship between irrigation amount, fertilizer rate, and yield.

We constructed a bubble chart to determine the optimal combination (Figure 2). As illustrated in the chart, the objective was achieved by applying 4322 m^3^ ha^−1^ of irrigation water and 225–135–270 kg N: P_2_O_5_: K_2_O ha^−1^.

### 2.6. Water and Fertilizer Management Based on Yield Using Grain Nitrogen Accumulation Correlation Analysis

**Figure 3:**
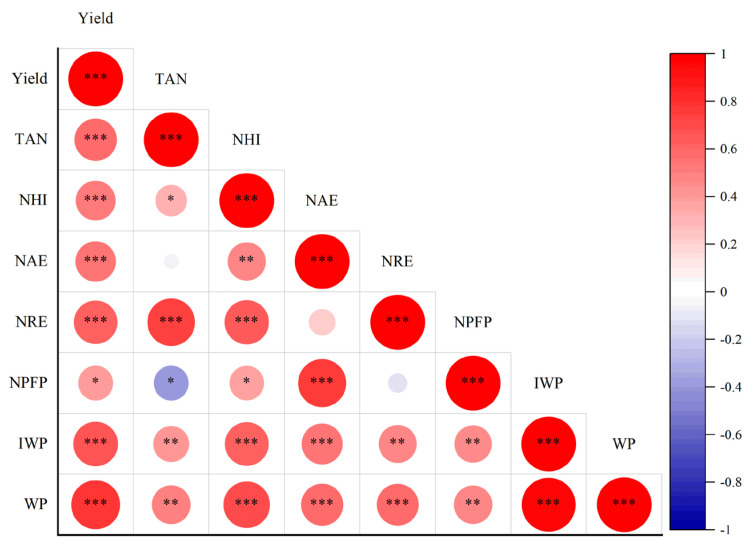
Water and fertilizer management based on yield using grain nitrogen accumulation correlation analysis., and significant treatment effects within a main category are denoted by * (0.01 < *p* ≤ 0.05), ** (*p* ≤ 0.01) or *** (*p* ≤ 0.001).

We constructed a correlation matrix to compare the correlations between all indicators (Figure 3). As illustrated in the figure, NHI, TAN, NAE, NPFP, NRE, WP, and IWP exhibit significant correlations with yield. NHI, TAN, NAE, NPFP, NRE, and yield significantly correlate with WP.

## 3. Discussion

### 3.1. The Relationship between Rice Yield and the Absorption of Nitrogen, Phosphorus, and Potassium in Rice Plants under the Interaction of Irrigation and Fertilizer Rice Plants under the Interaction of Irrigation and Fertilizer

Sun [26] reported that there is a significant interaction between water and nitrogen that affects the accumulation, translocation, allocation, and yield of nitrogen, phosphorus, and potassium during the main growth stages of rice. This water–nitrogen interaction demonstrates a significant or highly significant positive correlation with the absorption, and translocation of nitrogen, phosphorus, and potassium, as well as with yield, across various growth stages. We discovered that the alterations in fertilizer application rates beneath diverse irrigation methods continuously restricted the overall accumulation of nitrogen, phosphorus, and potassium in rice plants, as well as the translocation of these nutrients from vegetative organs during the fruiting period. Remarkably, there was a notable consistency in the changes noticed for nitrogen, phosphorus, and potassium throughout different growth stages under various water and fertilizer management practices in both the experimental grains. In addition, the correlation analyses of nitrogen, phosphorus, and potassium during the two-year trial period disclosed significant or highly significant correlations between different irrigation methods and fertilizer application rates regarding the absorption and accumulation of these nutrients in each growth stage. This implies that the interaction between water and fertilizer can also facilitate the synergistic uptake of nutrients in rice. This finding validates and supplements previous research, further highlighting that capitalizing on the advantages of the water–fertilizer interaction is fundamental for high-yield cultivation [27,28]. Some studies [29,30,31] reported that the total amount of nitrogen accumulation is different among different types and among different rice varieties of the same type, which in turn affects the yield. Sun [26] also clearly stated that under the interaction of different water management and nitrogen application rates, there is an extremely significant positive correlation between the total cumulative amounts of nitrogen, phosphorus, and potassium in rice plants and the yield. Our correlation analysis revealed a highly significant relationship between the total accumulation of nitrogen, phosphorus, and potassium in rice plants and the yield under different irrigation methods and fertilizer application rates. During the two-year experimental period, the W1 treatment enhanced the absorption, accumulation, and translocation of nitrogen, phosphorus, and potassium in various growth stages of rice, increasing the nutrient content in the grains. When combined with the F3 fertilizer application rate, it achieved the dual goals of increasing the yield and enhancing the nutrient use efficiency, establishing it as the optimal water–fertilizer coupling strategy in this study.

### 3.2. The Effect of Different Irrigation and Fertilizer Interactions on Rice Yield and Water–Fertilizer Use Efficiency

The water-saving irrigation techniques for rice, such as wet-shallow irrigation, alternate wetting and drying irrigation, controlled irrigation, and dry cultivation, have significantly increased water productivity [32,33,34]. However, the impact on yield varies depending on soil texture, soil drying level, and rainfall during the rice growing season [35]. Some studies have shown a decrease or no significant change, while others have demonstrated a decrease or no significant change [36,37]. The water-saving management technique employed in this study aligns with previous research findings regarding increasing rice yield. Its effect on water productivity is primarily achieved by reducing irrigation water input while enhancing yield. Research has indicated that wet-shallow irrigation can significantly enhance water productivity while reducing water consumption [38]. Xie [39] found that compared to conventional irrigation, wet-shallow irrigation led to a notable increase in water productivity by 11.3% despite a 24.3% reduction in irrigation water. Similarly, Zhang [40] reported that wet-shallow irrigation decreased irrigation water usage in paddy fields by 11.3–17.2% compared to shallow water irrigation while also increasing water productivity in rice irrigation by 3.2–30.0%. In our study, wet-shallow irrigation reduced irrigation water by 35.2% in 2022 compared to flooded irrigation, resulting in improvements of 42.0~42.8% and 25.7~25.9% in irrigation water productivity and total water productivity, respectively. In 2023, Similar results were also observed. These improvements might be attributed to the reduced irrigation volume and frequency associated with wet-shallow irrigation, which decreased water depth in the paddy fields, thus minimizing water leakage and evaporation [41]. Additionally, wet-shallow irrigation subjects rice to optimal stress during its growth cycle, promoting healthier growth and production, and ultimately enhancing rice yield and water productivity [42].

Within the fertilizer application range set in this study, the effect of increased fertilizer application on rice grain yield varies under different water management conditions. The trend of rice yield change is consistent in two years under water-saving conditions, with the highest yield achieved at the F3 treatment. In this study, the W1F3 treatment improved rice nitrogen (phosphorus, potassium) uptake, harvest index, and fertilizer use efficiency compared to other treatments. This indicates that wet-shallow irrigation improves rice fertilizer utilization efficiency, consistent with Sun’s [26] research results. This may be because wet-shallow irrigation reduces the water level in the paddy field, promotes root respiration, and enhances the absorption of nutrients, thereby improving rice fertilizer utilization efficiency [43]. Thus, high-quality hybrid rice varieties can achieve a synergistic efficiency of yield and water and fertilizer efficiency in intensive rice systems under a cultivation model combining lower fertilizer application (F3) with wet-shallow irrigation water management (W1).

## 4. Materials and Methods

### 4.1. Overview of Study Site

**Figure 4:**
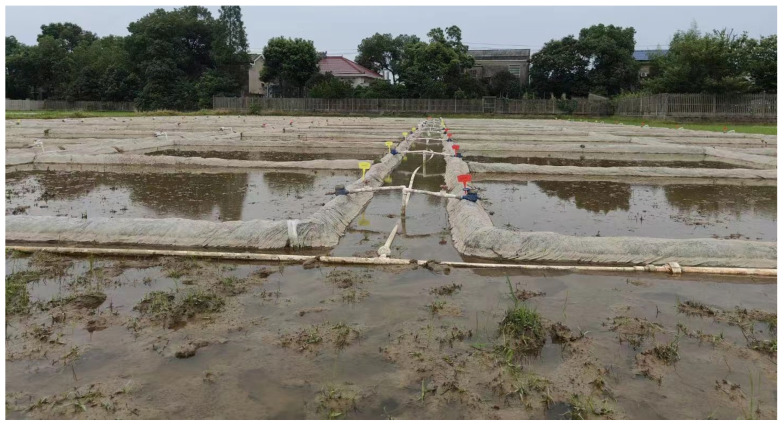
Water pipes and water meters in the community during the early stage of the experiment.

The experiment took place in the rice fields of farmers located in Oujiangcha village (coordinates: 28°29′55″ N 112°35′59″ E, 12 m a.s.l.), Oujiangcha town, Yiyang city, during the years 2022 and 2023. Table 6 presents the chemical properties of the soil in the 0 to 20 cm layer of the rice field prior to transplanting in 2022. This study conducted a split-split plot experiment with three factors: variety, water, and nitrogen fertilizer. The experiment comprised 72 split plots, each covering an area of 20 m^2^, resulting in a total experimental area of 1440 m^2^ (excluding water channels). The main plots were assigned to water treatments (W), with three levels: W1—continuous wet-shallow irrigation throughout the growth period, W2—flooded irrigation, and W3—thin, shallow, wet, dry irrigation. The water layer control standards for these three irrigation modes are summarized in Table 7. All three irrigation treatments involved manual watering, with a precise calculation of the irrigation volume using water meters (Figure 4). The split plots were designated for variety (V), featuring two levels: Xin Xiang Liang You 1751 (V1), provided by Hunan Jinse Nonghua Seed Industry Technology Co., Ltd. (Changsha, China), and Yue Liang You Meixiang Xin Zhan (V2), supplied by Yuan Longping High-Tech Agriculture Co., Ltd. (Changsha, China).

The split-split plots focused on nitrogen application (N) with four levels, N1, N2, N3, and N4, corresponding to pure nitrogen applications of 0, 180, 225, and 270 kg ha^−1^. The basic seedling rate was set at three seedlings, with a transplanting density of 225,000 hills per hectare. Nitrogen fertilizer (urea) was applied in a ratio of base fertilizer/tiller fertilizer/ear fertilizer = 5:3:2. The base fertilizer, tiller fertilizer, and ear fertilizer were applied 1–2 days before transplanting, 10 days after transplanting, and at the beginning of young ear differentiation (jointing stage), respectively. A consistent NPK ratio of N: P_2_O_5_: K_2_O = 1: 0.6: 1.2 was used for all fertilizer applications, with all of the phosphorus fertilizer (P_2_O_5_, 16%) applied as the base fertilizer and potassium fertilizer (K_2_O, 60%) split evenly between the base and tiller fertilizers. The rigorous management of pests, diseases, and weeds follows the principles of high-yield cultivation.

Three continuous data in the table (e.g., 0–20–30) represent the lower and upper limits of irrigation and the maximum limit after rain, respectively; RS represents the returning green stage, ES represents the early tillering stage, LS represents the late tillering stage, JS represents the jointing and booting stage, and HS represents the heading stage. GS represents the grain-filling stage, and MS represents the maturity stage.

### 4.2. Sampling and Measurement

**Figure 5:**
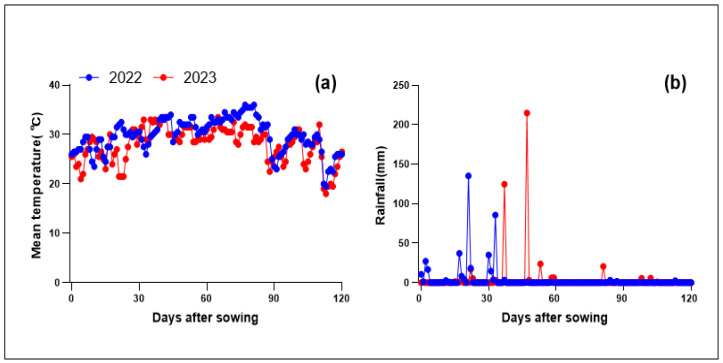
Daily mean temperature (**a**) and daily cumulative rainfall (**b**) during rice-growing season in 2022 and 2023.

The meteorological data, including minimum temperature, maximum temperature, and precipitation, was sourced from the local meteorological bureau (Figure 5). Irrigation water productivity (IWP) is the amount of dry matter produced per unit volume of water (m^3^) irrigated in a 1-hectare rice field, representing the production efficiency of irrigation water. Total water productivity (WP), on the other hand, refers to the amount of dry matter produced per unit volume of water (m^3^) consumed in a 1-hectare rice field, indicating the overall efficiency of water utilization [44].

At the heading and maturity stages, 10 representative rice plants were selected based on the average number of panicles per plot. These plants were thoroughly washed with water, their roots were trimmed, and they were manually threshed. The plant materials were then separated into three parts: straw, filled grains, and empty grains. The samples were first killed at 105 °C for 30 min and then dried to a constant weight at 80 °C before being weighed. A plant grinder was used to pulverize the plant samples to determine nitrogen, phosphorus, and potassium contents in the stems, leaves, and grains. Nitrogen was measured using the Kjeldahl method, phosphorus was determined by the molybdenum–antimony colorimetric method, and potassium was analyzed with a flame photometer [45].

At the maturity stage, one hundred effective rice spikes were meticulously examined in each plot. Subsequently, twelve hills were randomly chosen from each plot, taking into account the average number of effective spikes. These samples were then transported to the laboratory to investigate several key parameters, including panicle number × 104 ha^−1^, spikelet per panicle, spikelet filling, and grain weight. The entire plot of plants was harvested and dried separately for each plot, and the actual yield was calculated after adjusting the grain moisture content to 14% [46].

The plant’s total nitrogen (phosphorus, potassium) uptake is calculated as the sum of the nitrogen (phosphorus, potassium) absorbed by the stem, leaves, and grains. The nitrogen (phosphorus, potassium) harvest index (HI) is calculated as the ratio of grain nitrogen (phosphorus, potassium) uptake at maturity to the total nitrogen (phosphorus, potassium) uptake of the plant. Nitrogen (phosphorus, potassium) recovery efficiency (RE) represents the percentage difference between the accumulation of nitrogen (phosphorus, potassium) in plants from treated and untreated areas relative to the amount of nitrogen (phosphorus, potassium) applied. Nitrogen (phosphorus, potassium) partial factor productivity (PFP) is determined by dividing the yield in the treated area by the amount of nitrogen (phosphorus, potassium) applied. Agronomic efficiency (AE) of nitrogen (phosphorus, potassium) fertilizer refers to the increase in rice yield per unit of nitrogen (phosphorus, potassium) applied, calculated as the difference in rice yield between treated and untreated areas divided by the level of nitrogen (phosphorus, potassium) applied [47].

### 4.3. Data Analysis

The data were analyzed using analysis of variance (ANOVA) conducted in SAS Version 9.1.2. Means of fertilizer level, irrigation amount, and variety treatments were compared using the least significant difference test (LSD) at a 0.05 probability level. Graphs were constructed using Microsoft Excel 2017 (Microsoft Corp., Redmond, WA, USA).

## 5. Conclusions

In a two-year field study, we have determined the interactive effects of irrigation and fertilization on rice yield. Across different varieties, irrigation and fertilization significantly enhanced grain yield and the absorption of nitrogen, phosphorus, and potassium absorption. By combining reasonable irrigation methods with nitrogen management, we observed improvements in yield, water productivity, and fertilizer utilization efficiency. Therefore, we recommend using wet-shallow irrigation and appropriate fertilization (N- P_2_O_5_-K_2_O: 225–135–270 kg ha^−1^) in rice cultivation, especially in the middle and lower reaches of the Yangtze River, to enhance rice yield and water and fertilizer utilization efficiency. The combined application of wet-shallow irrigation and adequate fertilization achieves “compensating water with fertilizer” and “regulating water with fertilizer”, potentially contributing to the synergistic efficiency of yield, water, and fertilizer in intensive rice systems. In the future, further research can develop real-time sensors to measure the soil moisture and nutrient levels, so as to achieve more adaptive and targeted irrigation and fertilization.

## Figures and Tables

**Table 1:** Fertilizer, irrigation, variety, and year affect grain yield and yield components. No significant four-way interactions were observed, and only two three-way interactions (F × V × Y and V × W × Y) were significant for rice yield properties. Variables for which the model simplification process eliminated the non-significant (NS) three-way interactions are marked as NS. Significant treatment effects within a main category are denoted by * (0.01 < *p* ≤ 0.05) or ** (*p* ≤ 0.01).

SOV	Yield	Panicles (×10^4^ ha^−1^)	Spikelet’s Panicle^−1^	Spikelet Filling (%)	Grain Weight (mg)
F	**	**	**	**	**
V	**	**	**	**	**
W	**	**	**	**	*
Y	**	**	**	**	**
F × V	**	**	**	**	NS
F × W	**	NS	NS	NS	NS
F × Y	**	NS	NS	**	NS
V × W	NS	**	NS	**	NS
V × Y	**	NS	NS	NS	NS
W × Y	NS	NS	NS	**	NS
F × V × Y	NS	NS	NS	**	NS
V × W × Y	NS	NS	NS	**	NS

**Table 2:** The absorption capacity of nitrogen by the aboveground part of hybrid rice plants (kg ha^−1^).

Item	Treatment	Heading		Mature		
		Straw	Grain	Straw	Grain	Total
Year	2022	125.8 b	19.96 a	54.84 b	111.73 b	166.57 b
	2023	132.72 a	21.66 a	58.51 a	115.58 a	174.53 a
Variety	XXLY1751	129.95 a	22 a	57.22 a	117.34 a	174.56 a
	YLYMXXZ	127.37 a	19.78 b	55.75 b	110.62 b	166.37 b
Strategies	W1F1	75 f	17.48 ef	36.35 e	74.47 g	110.81 f
	W1F2	127.05 d	19.61 cd	57.03 d	123.03 d	180.06 d
	W1F3	149.52 c	23.34 b	62.22 bcd	143.89 a	206.11 a
	W1F4	165.86 b	26.59 a	67.57 abc	136.63 b	204.2 ab
	W2F1	72.24 f	15.54 g	33.58 e	68.3 h	101.88 g
	W2F2	118.74 e	18.28 def	58.99 d	110.9 f	169.89 e
	W2F3	144.53 c	20.42 c	62.56 bcd	128.27 cd	190.83 c
	W2F4	176.71 a	25.17 a	68.26 ab	125.18 cd	193.44 c
	W3F1	75.93 f	16.67 fg	36.97 e	72.82 gh	109.79 f
	W3F2	126.78 d	18.95 cde	59.47 d	117.03 e	176.5 d
	W3F3	146.86 f	22.42 b	65.62 abc	136.89 b	202.5 ab
	W3F4	164.66 f	26.23 a	69.23 a	130.37 c	199.6 b
SOV	F	**	**	**	**	**
	W	**	**	**	**	**
	V	**	**	**	**	**
	Y	**	**	**	**	**
	F × W	**	NS	**	**	**
	F × W × V	NS	NS	NS	NS	NS
	F × W × Y	*	NS	NS	NS	NS
	F × W × V × Y	NS	NS	NS	NS	NS

Within a column for each year, means followed by the same letters are not significantly different according to LSD (0.05). SOV, source of variation, and significant treatment effects within a main category are denoted by * (0.01 < *p* ≤ 0.05) or ** (*p* ≤ 0.01).

**Table 3:** The absorption capacity of phosphorus by the aboveground part of hybrid rice plants (kg ha^−1^).

Item	Treatment	Heading		Mature		
		Straw	Grain	Straw	Grain	Total
Year	2022	20.06 b	6.27 b	8.26 b	27.9 b	36.15 b
	2023	23.16 a	6.66 a	9.77 a	30.6 a	40.37 a
Variety	XXLY1751	21.72 a	6.84 a	9.08 a	30.58 a	39.66 a
	YLYMXXZ	21.44 a	6.12 b	8.89 a	28.29 b	37.17 b
Strategies	W1F1	14.51 g	4.4 g	5.72 e	24.61 f	30.33 a
	W1F2	20.99 ef	6.35 d	8.62 d	27.45 e	36.06 a
	W1F3	24.67 bc	7.78 a	9.69 bcd	37.63 a	47.32 b
	W1F4	25.67 b	7.53 bc	10.89 ab	31.96 c	42.85 c
	W2F1	14.23 g	4.6 f	6.06 e	22.78 g	28.84 c
	W2F2	19.8 f	6.22 de	8.7 cd	25.15 f	33.85 d
	W2F3	23.09 cd	7.64 ab	9.6 bcd	35.04 b	44.65 e
	W2F4	27.42 a	7.57 bc	11.22 a	29.98 d	41.2 e
	W3F1	15.18 g	4.54 fg	6.13 e	24.01 fg	30.14 f
	W3F2	21.72 de	6.12 e	9.24 cd	26.77 e	36.01 g
	W3F3	25.28 b	7.53 bc	10.48 ab	36.68 a	47.16 gh
	W3F4	26.39 ab	7.45 c	11.45 a	31.16 cd	42.61 h
SOV	F	**	**	**	**	**
	W	**	**	**	**	**
	V	*	**	**	**	**
	Y	**	**	**	**	**
	F × W	**	**	**	NS	NS
	F × W × V	NS	**	NS	NS	NS
	F × W × Y	NS	*	NS	NS	**
	F × W × V × Y	NS	NS	NS	NS	NS

Within a column for each year, means followed by the same letters are not significantly different according to LSD (0.05). SOV, source of variation, and significant treatment effects within a main category are denoted by * (0.01 < *p* ≤ 0.05) or ** (*p* ≤ 0.01).

**Table 4:** The absorption capacity of potassium by the aboveground part of hybrid rice plants (kg ha^−1^).

Item	Treatment	Heading		Mature		
		Straw	Grain	Straw	Grain	Total
Year	2022	172.96 b	6.27 b	140.22 b	27.9 b	36.15 b
	2023	193.46 a	6.66 a	150.90 a	30.6 a	40.37 a
Variety	XXLY1751	188.68 a	6.84 a	155.01 a	30.58 a	39.66 a
	YLYMXXZ	178.49 b	6.12 b	136.36 b	28.29 b	37.17 b
Strategies	W1F1	132.05 g	9.51 g	96.98 h	15.45 g	112.44 h
	W1F2	187.2 e	13.46 de	141.83 e	23.43 d	165.26 e
	W1F3	207.26 c	16.9 c	168.36 b	29.26 a	197.63 b
	W1F4	222.09 a	21.2 b	169.76 b	28.26 bc	198.02 b
	W2F1	127.06 h	9.98 f	105.85 f	16.31 f	122.16 f
	W2F2	180.58 f	13.78 d	145.88 d	22.88 de	168.76 d
	W2F3	201.97 d	16.74 c	166.94 b	28.69 ab	195.63 b
	W2F4	215.13 b	21.78 a	174.57 a	28.41 bc	202.98 a
	W3F1	128.26 h	9.38 g	102.23 g	16.03 fg	118.26 g
	W3F2	182.56 f	13.27 e	142.9 e	22.5 e	165.4 e
	W3F3	203.75 d	16.66 c	163.28 c	28.25 bc	191.53 c
	W3F4	215.11 b	20.91 b	169.62 b	27.92 c	197.54 b
SOV	F	**	**	**	**	**
	W	**	**	**	**	**
	V	**	**	**	**	**
	Y	**	**	**	**	**
	F × W	**	**	**	**	**
	F × W × V	**	**	**	**	**
	F × W × Y	**	**	**	*	**
	F × W × V × Y	**	NS	**	NS	**

Within a column for each year, means followed by the same letters are not significantly different according to LSD (0.05). SOV, source of variation, and significant treatment effects within a main category are denoted by * (0.01 < *p* ≤ 0.05) or ** (*p* ≤ 0.01).

**Table 5:** Agronomic N use efficiency (NAE), partial factor productivity of applied N (NPFP), apparent recovery efficiency of N (NRE), and N harvest index (NHI) under different irrigation and fertilizer treatments in 2022 and 2023.

Item	Treatment	AE	PFP	RE	HI
Year	2022	8.94 a	37.67 b	36.12 a	67.02 a
	2023	10.39 a	39.14 a	36.45 a	66.37 a
Variety	XXLY1751	9.85 a	40.99 a	36.58 a	67.25 a
	YLYMXXZ	9.78 a	36.92 b	36.24 a	66.4 b
Strategies	W1F1				67.12 bcd
	W1F2	10.55 a	47.13 a	34.72 b	68.32 ab
	W1F3	12.15 a	41.4 c	42.35 a	69.82 a
	W1F4	6.96 b	31.35 e	34.74 b	66.93 bcd
	W2F1				67.01 bcd
	W2F2	10.83 a	45.77 ab	34.5 b	65.27 de
	W2F3	11.08 a	39.02 d	39.57 a	67.21 bcd
	W2F4	7.31 b	30.6 e	33.6 b	64.69 e
	W3F1				66.29 cde
	W3F2	10.82 a	45.63 b	33.72 b	66.29 cde
	W3F3	11.71 a	39.56 d	41.21 a	67.6 bc
	W3F4	6.91 b	30.12 e	33.26 b	65.32 de
SOV	F	**	**	**	**
	W	**	**	**	**
	V	NS	**	NS	**
	Y	NS	**	NS	NS
	F × W	**	**	**	**
	F × W × V	NS	NS	NS	NS
	F × W × Y	NS	NS	NS	NS
	F × W × V × Y	NS	NS	NS	NS

Within a column for each year, means followed by the same letters are not significantly different according to LSD (0.05). SOV, source of variation, and significant treatment effects within a main category are denoted by ** (*p* ≤ 0.01).

**Table 6:** Soil pH, organic matter, and total nitrogen and available nutrients (N, P, K) of the experimental field.

PH	Organic Matter (g kg^−1^)	Total Nitrogen (g kg^−1^)	Olsen Phosphorus (mg kg^−1^)	Alkali-Hydrolysis Nitrogen (mg kg^−1^)	Exchangeable Potassium (mg kg^−1^)
5.1	34.8	1.6	13.8	166.8	106.4

**Table 7:** Thresholds of water level for different rice irrigation modes.

Irrigation	RS	ES	LS	JS	HS	GS	MS
W1	0–20–30	0–20–30	SF	0–20–30	0–20–30	0–20–30	DF
W2	20–50–70	20–50–70	SF	20–50–70	20–50–70	20–50–70	DF
W3	10–30–50	10–30–50	SF	10–40–60	10–40–60	10–20–40	DF

## Data Availability

The data presented in this study are available upon request from the corresponding author.
